# Capacitive Photodetector Thin-Film Cells of Cu-As_2_S_3_-Cu as Revealed by Dielectric Spectroscopy

**DOI:** 10.3390/s22031143

**Published:** 2022-02-02

**Authors:** Paul Ganea, Gabriel Socol, Sorin Zamfira, Nicolae Creţu, Elena Matei, Adam Lőrinczi

**Affiliations:** 1 National Institute of Materials Physics, 405A Atomistilor St., RO-077125 Magurele, Romania; paul.ganea@infim.ro (P.G.); elena.matei@infim.ro (E.M.); 2 National Institute for Laser, Plasma and Radiation Physics, 409Atomistilor St., RO-077125 Magurele, Romania; gabriel.socol@inflpr.ro; 3 Department of Product Design, Mechatronics and Environment, Transilvania University of Braşov, 29 Eroilor Blvd., RO-500036 Brașov, Romania; zamfira@unitbv.ro (S.Z.); cretu.c@unitbv.ro (N.C.)

**Keywords:** a-As_2_S_3_, copper ions, dielectric spectroscopy, electrical conductivity, photodetector cell

## Abstract

The As_2_S_3_-Cu interface was studied by dielectric spectroscopy measurements on Cu-As_2_S_3_-Cu thin film heterostructure samples to assess the charge carriers’ contribution to the electrical properties of such an interface. Three-dimensional printed masks ensured good reproducibility during the PLD deposition of heterostructure samples. The samples were tested for electrical conductivity and AC photoconductivity by dielectric spectroscopy measurements. DC bias voltages and light were applied to the samples. The electrical capacity of the thin film heterostructure can be modified electrically and optically. We observed long-term photoconductivity with a time dependency that was not exponential, and a quick change of the electrical capacity, indicating the potential of the heterostructure cells as photodetector candidates.

## 1. Introduction

Amorphous As_2_S_3_ thin films have remarkable electrical and optical properties making them strong candidates for various applications. Scientific interest in researching them has remained strong decades after the first discoveries were made [1]. This composition of As-S has an optical absorption edge of around 500 nm and shows a high optical transmission in the infrared (IR) and mid-infrared (MIR) spectral domain [2]. From a structural point of view, a-As_2_S_3_ has a sparse network capable of accommodating certain ionic or atomic species [2].

In this paper, we study the modifications of the electrical properties of Cu-As_2_S_3_-Cu heterostructures. We wanted to test if light and an electric field applied to the heterostructure would cause diffusion of copper into the noncrystalline matrix of the a-As_2_S_3_ thin film or if the heterostructure would evoke an electronic response. We studied the behaviour of thin a-As_2_S_3_ film deposited onto a thin Ag layer illuminated with a 532 nm wavelength green laser light. These bi-layer structures changed their structural and optical properties upon illumination with the monochromatic green light given by laser diodes [2,3].

These changes are attributable to the diffusion of Ag species (ions and atoms) through the network of a-As_2_S_3_ film due to green-laser-light illumination. Since light is the propagation of an electromagnetic field, it would be interesting to understand if a purely electric field can cause changes in the structural, electrical, or optical properties of similar Cu-As_2_S_3_ heterostructures. A deeper understanding of purely electric-field-induced property changes might contribute to identifying new applications based on this phenomenon.

In many technological solutions, there is a tendency to substitute silver with copper. An example is the mixed ionic-electronic conduction of MIEC-type materials, which are constituents of phase-change memory (PCM) structures. Copper is less diffusive than silver, so it is better controlled in the fabrication process of microelectronic structures and devices. Burr et al. discuss this problem in a review dedicated to access devices for non-volatile crossbar memory arrays [4].

The structure of the a-As_2_S_3_ thin films has been extensively studied and modelled during the past decades [5,6,7,8,9,10,11,12]. It is considered an aggregate of building units and fragments at molecular level, resembling molecular glasses. The ability of arsenic to shift from an electropositive state to an electronegative state, such as with oxo-anions and the metal arsenide, is a consequence of the electron occupation of bonding and antibonding orbitals of arsenic and its multiple ligands. Thus, we assumed a hypothesis that the Cu^+^ ions that form along the Cu-As_2_S_3_ interface will interact primarily with the interfaces of these structural building units [13].

In the case of samples with a complex composition and structure, electrical charge transport has multiple components, such as electronic conduction in bulk and through potential barriers, ionic conduction by hopping, or ionic conduction by skipping over the inter-grain potential barriers in disordered materials, etc.

The electrical properties of a given sample can be studied experimentally by broad-band dielectric spectroscopy (DS), which is equivalent to impedance spectroscopy (IS). This non-destructive method allows distinguishing between the different charge transport mechanisms or dielectric relaxation processes [14,15,16,17,18,19,20,21,22].

Two main polarization mechanisms may be studied by dielectric spectroscopy: the polarization due to the permanent molecular electrical dipoles and polarization given by the displacement and accumulation on certain surfaces of the mobile charge carriers. The displacement of charge carriers generates an electric current related to the electrical conductivity of the studied material. The magnitude of the conductivity depends on the extrinsic mobile charge carriers, the intrinsic mobile charge carriers, the material’s structure such as grain boundaries, electrostatic barriers at the molecular level, etc. [23,24,25].

## 2. Materials and Methods

For the deposition of the amorphous thin films, we used As_2_S_3_ pellets, with ~20 mm diameter and 3 mm thickness. The Cu thin layers were deposited from 99.9% purity Cu targets and were purchased from Sigma Aldrich. As deposition support, we used ordinary glass substrates, 1 mm thick and 25 × 25 mm size.

### 2.1. The 3D-Printed Mask Design

**Figure 1:**
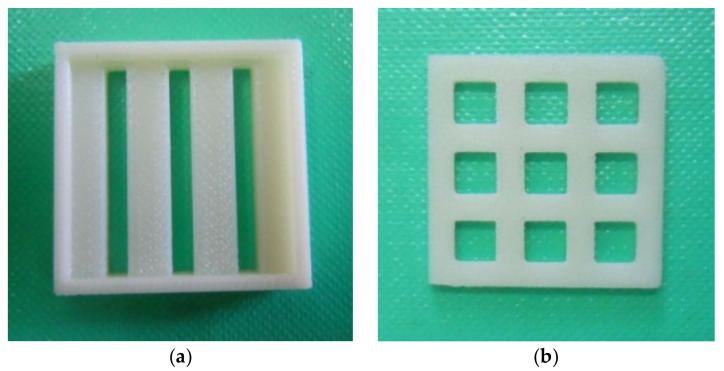
The 3D-printed masks.

To perform the sequence of the Cu, As_2_S_3_, and Cu heterostructure, we needed masks specially designed and produced for this process. After some initial tests with thin polymeric foils, we decided to use 3D-printed masks, which ensured excellent geometrical reproducibility and sharply defined edges of the deposited multilayer structure. An up-to-date description of this technique was given by Allen et al. [26]. Each horizontal and vertical slits width was 3 mm, the Cu mask had a length of 25 mm as in Figure 1a, and the mask for the As_2_S_3_ had nine square-shaped holes with 4 mm edge length each (Figure 1b).

### 2.2. Deposition of Thin-Film Cu-As_2_S_3_-Cu Heterostructures

The Cu and As_2_S_3_ thin films were deposited by pulsed laser deposition (PLD) using a KrF* excimer laser (λ = 248 nm and τ_FWHM_ = 25 ns) at a repetition rate of 5 Hz and 1.5 J/cm^2^ laser fluence. During the deposition, the pressure of the residual vacuum was 10^−3^ Pa, while the target–substrate distance was 5 cm.

The deposition of the successive layers to form the thin film Cu-As_2_S_3_-Cu heterostructures was conducted in three steps. In the first step, the three bottom Cu electrodes of the heterostructure were deposited. The electrodes were stripes the full length of the substrate (25 mm), with a width of 3 mm.

**Figure 2:**
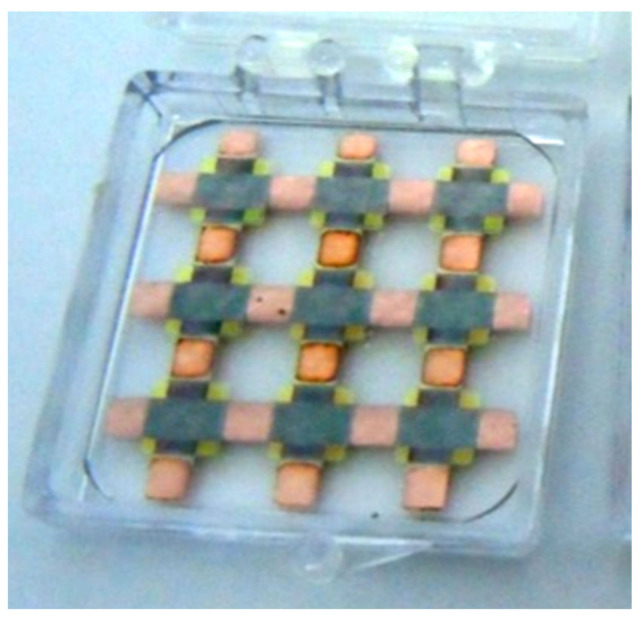
The heterostructure.

In the second step, we deposited As_2_S_3_ over three square zones of each electrode stripe, for a total of 9 squares with the edges of about 4 mm. For the third step, we deposited the set of three top Cu electrode stripes perpendicular to the bottom ones, having the square As_2_S_3_ spots as insulating islands in the intersection area, with the same 3 mm thickness, as the bottom Cu electrode stripes. In this way, we formed nine Cu-As_2_S_3_-Cu heterostructures of thin films, each of them electrically addressable individually, through the terminations of the bottom and top Cu electrodes, on the perpendicular sides of the glass substrate (1,1; 1,2; 1,3; 2,1; and 3,3), just like the elements of a 3 × 3 matrix (Figure 2).

The Cu-As_2_S_3_-Cu thin film heterostructures were investigated by two techniques. Firstly, we used scanning electron microscopy (SEM) to characterize the morphology of the cross-sections’ surface and gauge the thickness of the deposited thin films that build up the heterostructure. Secondly, we used dielectric spectroscopy (DS), which showed us the electrical behaviour of the Cu-As_2_S_3_-Cu structure, with and without an external voltage applied onto the structure. Thus, we measured the heterostructure’s impedance for a broad frequency range (from 10 Hz to 10 MHz) in the cases of 0 and 5 V external voltages applied. Both direct and inverse bias voltages were applied on the external Cu contacts of the heterostructure.

The impact of the light’s presence on values of the resistance and the heterostructure capacity was characterized by the transient *R*(t) and *C*(t) measurements during an off-on-off ambient light cycle. Each type of DS experiment was repeated at least once after 24 or 48 h to check the reproducibility of the sample’s behaviour. The term light refers to the visible range of the electromagnetic radiation spectrum, perceptible to the human eye, consisting of electromagnetic radiation with wavelengths between 380 nm (violet) and 760 nm (red). As a light source, we used a halogen bulb for lighting the rooms

SEM images were recorded with an EVO 50 XVP system from Zeiss (Jenna, Germany).

Dielectric spectroscopy measurements were performed with a broadband dielectric spectrometer, model Alpha-A High Performance Frequency Analyzer from Novocontrol (Montabaur, Germany).

## 3. Results and Discussion

### 3.1. Morphology Studies by Scanning Electron Microscopy (SEM)

The SEM images confirmed that the designed heterostructure was successfully realized. To observe the morphology of the cross-section’s surface by SEM, the cross-section was previously coated with an ultrathin (some nanometers thick) Au layer, to allow draining of the electric charge accumulated by the scanning e-beam.

**Figure 3:**
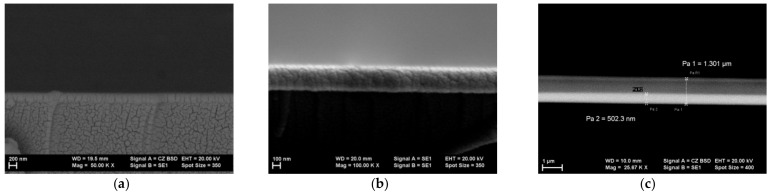
(**a**) As_2_S_3_ thin film on the substrate; (**b**) Cu thin film on glass substrate; (**c**) heterostructure on the substrate.

We observed a different structuring of the sample substrate’s glass compared to the As_2_S_3_ amorphous thin film, which shows a noticeable columnar feature (Figure 3a).

On the top of the structure, we have a uniform and dense Cu thin film but with some granular structural packing features (Figure 3b).

Figure 3c shows the cross-section of the heterostructure using backscattered electron analysis, which allowed us to estimate the thicknesses of the individual thin films composing the Cu-As_2_S_3_-Cu heterostructure: the bottom Cu electrode (~500 nm) had a comparable thickness to the a-As_2_S_3_ layer (~650 nm), while the top Cu electrode was much thinner (~150 nm). The thickness of the whole structure was about 1300 nm.

### 3.2. Dielectric Spectroscopy (DS) Measurements

DS measurements made in the presence of a static polarization voltage allow to suppress or emphasize a conduction mechanism, thus helping to distinguish between them.

For the impedance measurements, we designed experiments where a static polarization voltage was applied simultaneously with an alternative test voltage on bulk samples [27,28,29,30,31], as well as on thin-film structures with various junctions [32,33,34].

In order to see the behaviour of the Cu/As_2_S_3_ interface as well as that of the a-As_2_S_3_ thin film between the bottom and top Cu electrodes, the sample was contacted with thin Cu wires to a larger sample support, and provided with millimetre-sized gold contacts on alumina surfaces. This way, we had a safer and easier method of handling the sample. To connect it to the dielectric measurement equipment, a high-resolution Alpha analyser (Novocontrol) was used to measure the complex impedance, $Z\left(\omega \right)\;=\;Re\left(Z\left(\omega \right)\right)\;+\;i\;Im\left(Z\left(\omega \right)\right)\;=\;Z^{\prime }\left(\omega \right)\;+\;iZ^{\prime }\left(\omega \right)$.

Measurements were performed in the frequency range of 10–10^7^ Hz. During the dielectric spectroscopy measurements of the Cu-As_2_S_3_-Cu heterostructure, we measured the impedance versus the frequency of the external test signal, which had an amplitude of *u* = 20 mV.

The externally applied DC voltage (U_DC_) was gradually increased from 0 to U_DCmax_ (U_DCmax_ = 10 V) in steps of 0.25 V, followed by a decrease to −U_DCmax_ with the same voltage steps, in 3¼ cycles, obtaining a series of impedance spectra shown in Figure 5a,b.

In order to see the variation with time of the components of the impedance (i.e., the resistance and the capacitance) under the presence of light or an external bias voltage, we performed measurements in a transient regime, *Z*(t), in several situations: (a) with U_DC_ switched on/off, we first applied U_DC_ = 10 V external DC voltage; (b) with the light switch on/off, the sample was illuminated with several types of ordinary sources of light in the absence (U_DC_ = 0 V) and in the presence of a polarization voltage (U_DC_ = 10 V).

**Figure 4:**
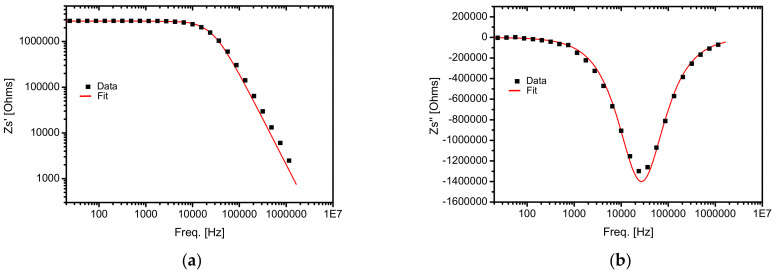
(**a**) Real part of impedance, *Z*′, versus the frequency (f) at U_DC_ = 10 V, t = 6.000 s, without light; (**b**) imaginary part of impedance, *Z*″, versus the frequency (f) at U_DC_ = 10 V, t = 6.000 s, without light.

From this large series of spectra, we selected two representative pairs (*Z*′, *Z*″) indicated with black data points in Figure 4a, $Z^{\prime }=Z^{\prime }\left(f\right)$ and Figure 4b, $Z^{\prime \prime }=Z^{\prime \prime }\left(f\right)$.

**Figure 5:**
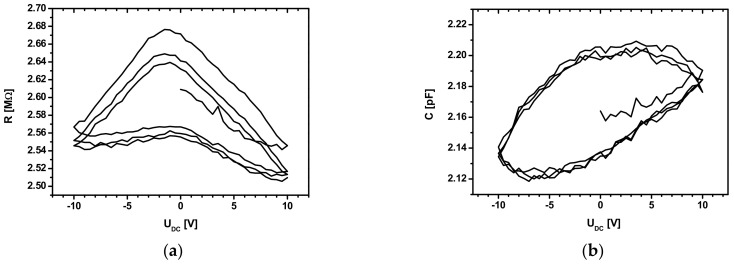
(**a**) *R* = *R*(U_DC_). Cycles start at 0 V and stop at 10 V polarization, after 3¼ cycles; (**b**) *C* = *C*(U_DC_). Cycles start at 0 V and stop at 10 V polarization, after 3¼ cycles.

As the duration of the measurement, we chose t = 6.000 s, during which the sample was subjected simultaneously to a continuous external bias voltage of U_DC_ = 10V and ambient light switched off-on-off for 1–2 h per interval, as shown in Figure 5.

In a double-logarithmic representation, the real component *Z*′ showed a linear decrease with frequency in the 10^4^–10^7^ Hz frequency range, while the imaginary component *Z*″ showed a minimum value around 20–30 kHz in a semi-logarithmic representation.

The shape of the spectra (Figure 4a,b) indicates that the measured cells had two main electric components: the resistor, *R*, and the capacitor, *C*, in a parallel group, for one single dielectric relaxation process. The impedance of an equivalent RC parallel connection is given by

(1) $$Z^{-1}\left(\omega \right)\;=\;R^{-1}\;+\;i\omega C$$

or:

(2) $$Z\left(\omega \right)\;=\;R/\left(1\;+\;i\omega \tau_{0}\right)$$

where ω = 2π⋅f is the angular frequency, *f* is the frequency, and $\tau_{0}\;=\;C\cdot R$ is the characteristic time. Fitting the experimental data with this function (Equation (1) or Equation (2)), we can obtain the real and the imaginary components of the equivalent circuit, which are *R* and *C,* respectively. An important fitting parameter is the characteristic time, $\tau_{0}\;=\;C\cdot R$, which is an intrinsic feature of the structure, since it does not depend on the sizes of the measured structure, such as layer thickness or sample surface area. For example, Figure 4a,b shows the fitting function for Equation (1) with a continuous red line.

As we may observe further on, all these three quantities, which appear in the impedance fitting function, are dependent on the direct voltage and the illumination applied on the studied samples. 

#### 3.2.1. Effect of DC Voltage Polarization Cycles

For the first type of experiments, we conducted several DC polarization cycles, with U_DC_ voltage having values between 10 and −10 V. In this case, the components of the impedance are shown as *R* in Figure 5a and as *C* in Figure 5b.

Figure 5a,b shows the dependence of resistance and cell capacity on the applied voltage between −10 and +10 V. One may remark a hysteresis-like effect of the electrical resistance, *R*, and of the capacity, *C*.

From the analysis of Figure 5a,b, the resistance, *R*, has a more significant dispersion, and the capacity, *C*, has a lower dispersion and a greater stability from one cycle to another. The cycle’s dispersion indicates a partial reversibility due to a high relaxation time. The curves in Figure 5a are somewhat symmetrical to the voltage polarity, which does not indicate the presence of a space-charge region at the Cu-As_2_S_3_ interfaces. The resistance shows an almost linear decreasing tendency versus the increasing direct voltage’s absolute value, suggesting a different mechanism contributing to the AC-DC field-effect conductivity.

To the best of our knowledge, the shape of the *R*–V and *C*–V characteristics given in Figure 5a,b have not yet been reported.

#### 3.2.2. Effect of Illumination Cycles

**Figure 6:**
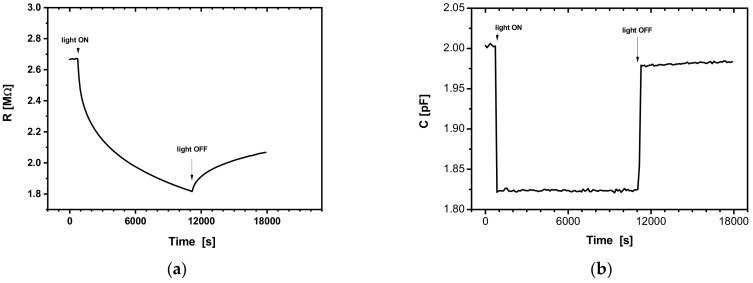
(**a**) *R*(t) at U_DC_ = 0 V, with light off-on-off; (**b**) *C*(t) at U_DC_ = 0 V, with light off-on-off.

**Figure 7:**
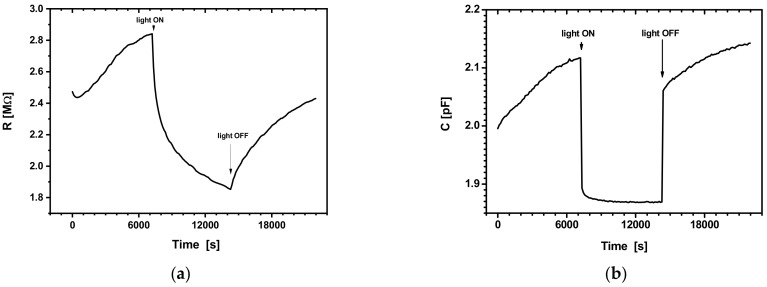
(**a**) *R*(t) at U_DC_ = 10 V, with light off-on-off; (**b**) *C*(t) at U_DC_ = 10 V, with light off-on-off.

On the other hand, the study of the photoconductivity assisted by an alternative electric field may be useful, since these measurements may yield information about the dynamics of the charge carriers, such as the relaxation time, for example. The results of transitory measurements in the presence of a U_DC_ = 0 V or U_DC_ = 10 V bias polarization, for an off-on-off ambient light, as used in the laboratory, are shown, respectively, in Figure 6a,b and Figure 7a,b.

In Figure 6a,b, one may observe two distinct domains for the time evolution of the electrical resistance and capacity, corresponding to some conduction mechanisms with different time constants. The large timescales involved may account for the remanence of the conductivity as well as for the hysteresis-like features in Figure 5a,b.

Unlike the M-S-M or M-I-M structures, which responded quickly to the application of light, in the case of the analysed structures here, the electrical resistance had a very long response time. In contrast, the capacity had a very short response time when the light was switched on/off. Upon switching the light on and off, the relative changes for the values of resistance and capacity were: ${\left.\left(\Delta R/R_{m}\right)\right|}_{U_{dc}}≅11.54\%$, ${\left.\left(\Delta R/R_{m}\right)\right|}_{Light}≅28.57\%$; ${\left.\left(\Delta C/C_{m}\right)\right|}_{U_{dc}}≅6.06\%$, ${\left.\left(\Delta C/C_{m}\right)\right|}_{Light}≅12.5\%$.

The slow decrease and increase in the photoconductivity, also known as long-term photoconductivity or persistent photoconductivity (PPC), is ascribed to potential barriers inside the sample called recombination barriers, which are related to different types of structural inhomogeneities [34,35,36,37,38,39,40].

We found experimentally that the long-term photoconductivity is characterized by an instantaneous relaxation time of the system’s photoresponse, given by

(3) $$\tau \;=\;\tau_{0}exp\left(E_{rec}/k_{B}T\right),$$

where $\tau_{0}$ may vary on a large scale between $10^{-12}\;\mathrm{and}\;10^{-4}\;s$, while the values of $E_{rec}$ are in the range of one-tenth of a millielectron volt. During the relaxation process, $E_{rec}$ decreases, the drop of the photoresponse is slower, and does not follow an exponential law [35].

PPC is caused by the steric separation of the non-equilibrium charge carriers by the electrical fields of the inhomogeneities; thus, their recombination will occur with a much smaller probability. The separation of the photogenerated charge carriers will create electrical fields that compensate for the fields of the inhomogeneities. Thus, the heights of the potential barriers decreases with the increase in the non-equilibrium charge carriers.

The most general form of inhomogeneity can be represented by a “random landscape” of the electrical potential, as in the case of the amorphous materials [36]. This random potential barrier system consists of two barriers: a recombination barrier ($E_{rec}$) and a drift barrier ($E_{drift}$). The minority charge carriers localize on the top of these barriers, while the main electrical current will flow through the valleys. In this way, to have a recombination process, the majority of charge carriers need to be activated to the maximum heights of the potential landscape, while for conduction, they need to be activated to the minimum heights. Thus, the random potential allows us to define the recombination barriers and the drift barriers in a unique way. The presence of a random potential landscape in samples with long-term photoresponse has been confirmed experimentally as well [37,38].

Considering that amorphous As_2_S_3_ thin films have their structure built up from several kinds of building units at the molecular level [5,6,7,8,9,10,11,12], similar to the molecular glasses, it seems plausible to understand the above electrical measurement results. In this heterostructure, we have a superposition of electrical charge distribution from the surface of the structure (from the contacts), as well as from the many internal interfaces of the structure. This charge distribution is also influenced by the polarization voltage applied to the heterostructure’s contacts.

The hysteresis-like feature in Figure 5a,b suggest the existence of a remanence for the electrical conductivity. One may note that on the return path, the values of the conductivity are larger compared to those measured on the direct path. The finding that the starting and final values of the conductivity are almost identical suggests that one may have a reversible component of the electrical charge diffusion.

From these results so far, we understand that the heterostructure’s response to the testing signals used is mainly electronic in nature, with a contribution of almost free Cu^+^ ions coming from the Cu/As_2_S_3_ interface. Large timescale behaviour supports this idea of having low-mobility charge carriers present in the structure. These charges are likely due to the electrons of the structure between the Cu layers. Additionally, some very loosely bonded Cu^+^ ions from the Cu-As_2_S_3_ interfaces may roam around and follow the electric field, giving rise to a reversible component of the Cu^+^ diffusion.

#### 3.2.3. Effect of Simultaneously Applied Light and Voltage

**Figure 8:**
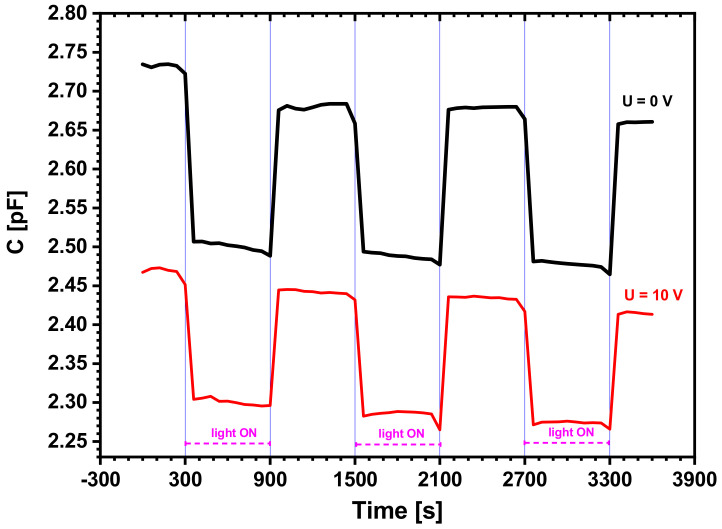
Capacity variation with illumination at polarization voltages of U_DC_ = 0 V and U_DC_ = 10 V.

To check the applicability of the structure as a light sensor, we also carried out two new series of measurements for the electrical capacity of the structure in two cases: (a) without polarization voltage on the contacts, and (b) with a U_DC_ = 10V polarization on the contacts (Figure 8).

We observed the quick and periodical change in the electrical capacity upon switching the illumination on and off in two situations: without polarization voltage (upper black graph) and with U_DC_ = 10 V polarization voltage (lower red graph). We found the bias voltage did not change the response shape, and the structure’s ability shifted the values with respect to the U_DC_ = 0 V case. The value of the electrical capacity dropped consistently upon illumination and recovered without illumination in both cases.

## 4. Conclusions

The AC photoconductivity and AC-DC electric field effect for a Cu-As_2_S_3_-Cu heterostructure with amorphous As_2_Se_3_ films were studied.

The setup built on 3D-printed masks essentially improved the quality of the thin-film structures deposited with them and ensured the reproducibility of the structure’s geometry.

Dielectric spectroscopy measurements revealed the frequency dependence for the impedance of the heterostructure in the *f* = 1 to 10^7^ Hz range.

The experimental results obtained on the basis of a large number of cycles for the polarization voltage allowed us to observe more complex dynamic phenomena with different temporal characteristics.

A hysteresis-like feature was evidenced for the impedance, $Z\;=\;Z\left(U_{\mathrm{DC}}\right)$, in cycles for $U_{\mathrm{DC}}$ = 0 to 10 V, indicating an electric charge diffusion.

The structures proved to be sensitive to an external direct-reverse voltage applied on the electrodes and to the on-off switching of the ambient light. These two external parameters, the voltage and the light, have opposite effects on the resistance and capacity of the heterostructure: they increase if an external voltage is applied, but they decrease upon the presence of light shining on the structure. The profile of the resistance change in time shows transitory behaviour with large characteristic time, while the capacity changes very quickly, almost instantaneously.

The transient increase and decrease in the photo-response are very slow and follow a non-exponential time dependence.

Based on these results, we suggest that the heterostructure’s electrical response to the testing voltages used is mixed, mainly of electronic nature, but with some possible contribution of almost free Cu^+^ ions originating from the Cu/As_2_S_3_ interface.

Because the presented structure’s electrical capacity varies very fast as light is turned on or off, the structure works as a photodetector. It may be suitable for applications where light sensing, i.e., photodetection, is needed. It may be applied for environment monitoring in various industrial processes, in security and surveillance applications, or in access control.

## Data Availability

The data presented in this study are available upon request from the corresponding author.
